# Oxytocin levels do not change around a meal and correlate with reward-driven caloric consumption in adults with obesity

**DOI:** 10.1111/dom.70340

**Published:** 2025-12-15

**Authors:** Marie-Louis Wronski, Regine Boutin, Clara O. Sailer, Francesca Galbiati, Anna Aulinas, Maged Muhammed, Kamryn T. Eddy, Jennifer J. Thomas, Liya Kerem, C. Sue Carter, Shawn Nazarloo, John M. Davis, James E. Blevins, Katherine Holman, Julia Gydus, Sarah E. Smith, Elisa Asanza, Franziska Plessow, Elizabeth A. Lawson

**Affiliations:** 1 Neuroendocrine Unit, Department of Medicine, Massachusetts General Hospital and Harvard Medical School, Boston, Massachusetts, USA; 2 Translational Developmental Neuroscience Section, Division of Psychological and Social Medicine and Developmental Neurosciences, Faculty of Medicine, Technische Universität Dresden, Dresden, Germany; 3 Section of Endocrinology, Department of Medicine, Temple University, Lewis Katz School of Medicine, Philadelphia, Pennsylvania, USA; 4 Division of Endocrinology, Diabetes and Hypertension, Department of Medicine, Brigham and Women’s Hospital, Harvard Medical School, Boston, Massachusetts, USA; 5 Department of Endocrinology, Hospital de la Santa Creu i Sant Pau, IR-Sant Pau, CIBERER Unidad 747, ISCIII, Barcelona, Spain; 6 Eating Disorders Clinical and Research Program, Department of Psychiatry, Massachusetts General Hospital and Harvard Medical School, Boston, Massachusetts, USA; 7 Division of Pediatric Endocrinology, Department of Pediatrics, Hadassah-Hebrew University Medical Center, Jerusalem, Israel; 8 Kinsey Institute, Indiana University, Bloomington, Indiana, USA; 9 Department of Psychology, University of Virginia, Charlottesville, Virginia, USA; 10 Department of Psychiatry, University of Illinois at Chicago, Chicago, Illinois, USA; 11 Department of Veterans Affairs Medical Center, VA Puget Sound Health Care System, Office of Research and Development Medical Research Service, Seattle, Washington, USA; 12 Division of Metabolism, Endocrinology and Nutrition, Department of Medicine, University of Washington School of Medicine, Seattle, Washington, USA; 13 Department of Psychiatry and Behavioral Sciences, Weill Institute for Neurosciences, University of California, San Francisco, California, USA

**Keywords:** appetite control, clinical trial, obesity care, weight control

## Abstract

**Background:**

Exogenous oxytocin (OT) reduces reward-driven food intake. Less is known about endogenous OT and eating behaviour. Preclinical studies suggest peripheral OT levels may reflect the opposite of central appetite-related OT activity. In healthy females, circulating OT declines after eating. Whether this pattern exists in obesity and how endogenous OT relates to hedonic eating remains unclear. We hypothesised that OT would decrease postprandially in adults with obesity and that higher OT exposure, reflecting lower central OT signalling, would correlate with greater reward-driven caloric intake.

**Methods:**

Sixty-one adults with obesity (56% female; age [mean ± SE] 33.55 ± 0.81 years; BMI 36.77 ± 0.62 kg/m^2^) consumed a standardised meal following an overnight fast. OT was measured in peripheral blood pre-meal and 30, 60, and 120 min post-meal. Area under the curve (AUC) was calculated to capture OT exposure. Postprandial hedonic eating drive was assessed via visual analogue scales (VAS) and Cookie Taste Test (CTT). Meal-related OT dynamics were analysed using linear mixed effects models, relationships between OT exposure and hedonic eating drive were examined with linear regression and mediation analyses.

**Results:**

OT levels did not change in response to the meal. Greater OT AUC was associated with reduced postprandial satisfaction (*p* = 0.008, *d* = 1.00) and higher CTT caloric intake (*p* = 0.036, *d* = 0.62). The relationship between OT AUC and CTT caloric intake was mediated by OT’s effect on postprandial satisfaction (*p* = 0.014, proportion mediated = 53.28%).

**Conclusions:**

In obesity, OT levels did not change postprandially. Greater OT exposure was linked to lower satisfaction and increased hedonic eating, suggesting dysregulated OT signalling in obesity potentially contributing to overeating.

## 1 | INTRODUCTION

Oxytocin (OT), a sexually dimorphic hormone best known for its role in parturition and lactation, is under investigation as an anti-obesity therapeutic due to its anorexigenic and weight loss effects in rodent and primate models.^1–7^ According to existing evidence, OT may preferentially reduce reward-driven food intake. In rodents, OT administered directly into the basolateral amygdala,^8^ the nucleus accumbens core,^9^ and the ventral tegmental area (VTA)^10^—key brain regions involved in hedonic eating—reduced sucrose intake. Systemic OT administration reduced consumption of a fructose-sweetened beverage in non-human primates.^3^ Conversely, the injection of an OT antagonist increased sucrose consumption in rodents.^11^ Furthermore, OT deficient mice overconsume sucrose,^12^ suggesting that endogenous OT inhibits consumption of sugar. In humans, intranasal (IN) OT likewise decreased caloric intake in fasted and fed states.^5,13–18^ A mechanistic study of men with overweight/obesity who viewed images of high-calorie foods (vs. objects) after receiving a single dose of IN OT (vs. placebo) showed that OT reduced functional magnetic resonance imaging (fMRI) activation of the VTA^19^ and decreased connectivity between the VTA and other brain areas involved in food motivation.^20^ IN OT also increased fMRI activation of brain regions responsible for cognitive control over eating promoted goal-directed decision making during a behavioural task^21^ in men with overweight/obesity. These neural mechanisms might underlie OT’s potential to improve control over reward-driven eating. In line with findings from neuroimaging, a single dose of IN OT led to a reduction in the consumption of palatable snacks in a fed state in normal-weight and overweight/obese men.^14,22^ While these data demonstrate that supraphysiologic OT modulates reward-driven eating, the role of endogenous OT on hedonic food consumption is less understood in humans.

There are few studies examining endogenous OT patterns in response to food intake. In rats, activation of OT neurons in appetite pathways resulted in suppression of posterior pituitary release of OT to the circulation,^23^ suggesting that peripheral OT levels (in response to feeding) may reflect the opposite of central appetite-related OT activity. Nonetheless, studies of rats showed that peripheral OT levels increased after feeding with chow^24^ and gavage with sweetened condensed milk.^25^ In contrast, serum OT levels decreased by approximately 20% after a standardised mixed meal in normal-weight, healthy females aged 10–45 years^26^ and by 24% in women aged 18–35 years with mean body mass index (BMI) 25 kg/m^2^.^27^ Postprandial circulating OT levels also decreased in females with low-weight eating disorders, particularly in those with atypical anorexia nervosa, who exhibit comparable psychopathology to full-syndrome anorexia nervosa despite having higher body weight.^28^ However, in women with full-syndrome anorexia nervosa, there was no change in OT levels in response to a meal,^28^ suggesting abnormal OT-appetite signalling. To date, there have been no studies that describe the secretory patterns of endogenous OT following a meal nor the relationship between endogenous OT exposure and hedonic appetite or eating in humans with obesity.

Here, we investigated circulating OT levels in response to a standardised meal in adults with obesity. We hypothesised that OT levels would decrease postprandially, consistent with meal-related OT patterns in healthy females^26,27^ as well as those with low-weight eating disorders.^28^ Furthermore, we hypothesised that higher peripheral OT levels around the meal—likely reflecting less central anorexigenic signalling—would be associated with greater hedonic appetite and eating behaviour in a fed state in adults with obesity. Given evidence of sex-dependent OT effects in recent literature—for instance, central OT administration led to greater anorexigenic activity in male than female rodents^29^—we also explored the influence of biological sex on OT levels in response to a meal and their relationship with hedonic eating drive.

## 2 | METHODS

### 2.1 | Study design and participants

We included baseline data from 61 adults with obesity, who participated in a randomised clinical trial (NCT03043053) investigating IN OT as a weight loss therapeutic^16,30^ and who had at least one OT measurement available. This trial recruited participants aged 18–45 years with BMI ≥30 kg/m^2^ who were willing to maintain their current lifestyles for the duration of the trial. Exclusion criteria comprised oral contraceptive use in female participants, recent changes in lifestyle, history of cardiovascular disease, comorbidities and/or medications that could affect body weight such as weight loss supplements, clinically significant gastrointestinal disorders, history of bariatric surgery (except gastric banding), HbA1c ≥6.5%, history of an eating disorder or psychosis (please consult the Supplemental Materials [SM 1] for further exclusion criteria). This study was approved by the Mass General Brigham Institutional Review Board and executed in accordance with the Declaration of Helsinki. Written informed consent was obtained from each study participant after full explanation of the purpose and nature of all study procedures.

### 2.2 | Study procedures

**FIGURE 1:**
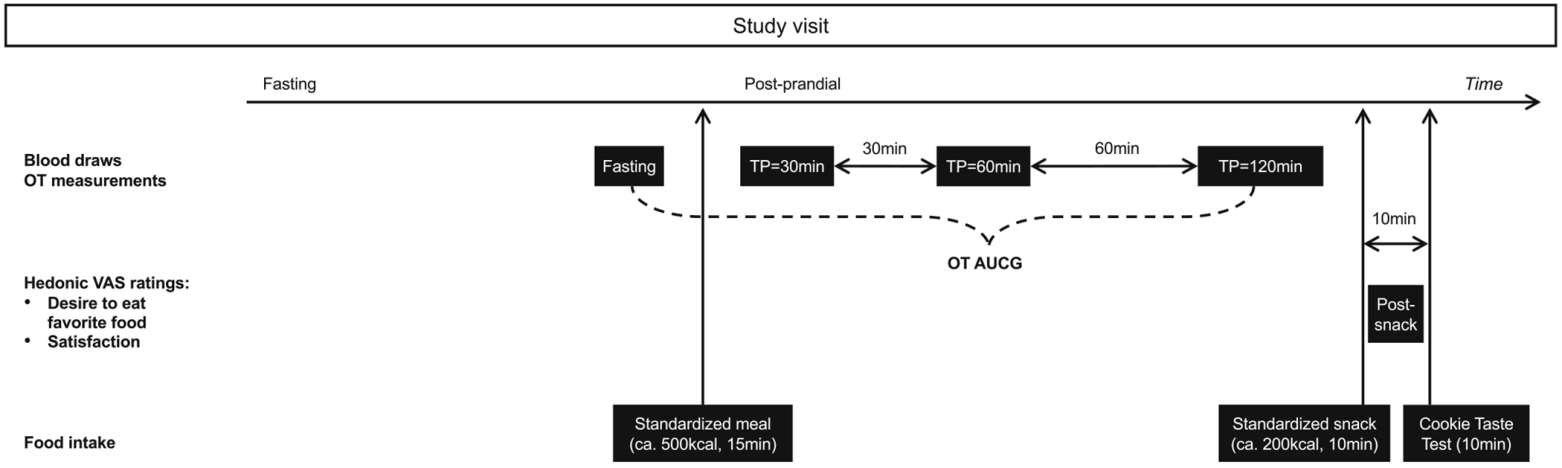
Procedures during the study visit. AUCG, area under the curve with respect to ground; OT, oxytocin; TP, time point; VAS, visual analog scale.

Participants were asked to fast for a minimum of 10 h prior to the study visit. At the beginning of the visit, medical history and physical examination were performed. BMI was calculated from height and weight measurements (weight in kg/[height in m]^2^). An intravenous line was placed, and fasting blood was drawn for OT measurement at approximately 8–9 a.m. after a minimum 10 h fast. Participants were then instructed to eat an approximately 500 kcal standardised mixed meal over 15 min. Participants selected between three meal options (option 1: cereal, milk, mini bagel with peanut butter; option 2: bagel, butter, milk; option 3: soy milk, cereal, gluten-free toast with margarine) of similar caloric and macronutrient content (approximately 60% carbohydrates, 20% fat, 20% protein). Subsequently, blood was drawn at 30, 60, and 120 min after the start of the meal (Figure 1). At 150 min after the start of the meal, participants had a standardised snack of their choice (option 1: vanilla yogurt/banana, option 2: applesauce/multigrain bar, option 3: apple slices/pretzels), consisting of approximately 200 kcal and similar macronutrient content (approximately 80% carbohydrates, 10% fat, 10% protein). The Cookie Taste Test (CTT)^31,32^ was performed 10 min after the standardised snack (Figure 1).

### 2.3 | OT measurement

Peripheral venous whole blood samples, collected into tubes with EDTA anticoagulant, were immediately put on ice, followed by centrifugation to separate plasma (at 4°C and 2800 rpm for 20 min). Plasma was then dispensed into aliquots and stored at −80°C until laboratory analysis. Plasma OT levels (time points [TP]: fasting; 30, 60, and 120 min after the standardised meal) were measured in batch in the laboratory of Drs. Nazarloo and Carter at the Kinsey Institute, Indiana University (Bloomington/IN) using a highly sensitive enzyme immunoassay (EIA; Arbor Assays/Ann Arbor/MI/USA; unextracted measurement). All samples were measured at the same time and in duplicate (mean values were used in analyses). The intra-assay coefficient of variance (CV) of OT measurements in our sample was computed as mean ± standard error (SE) = 8.97 ± 0.36%. Inter-assay CV of the OT assay was <10% according to the manufacturer. No participants were excluded based on CV values (all CV values were below the outlier/exclusion threshold: mean CV + 3*SD).

### 2.4 | Assessment of subjective appetite and hedonic eating behaviour

Visual Analog Scales (VAS) were used to assess momentary subjective homeostatic and hedonic appetite.^33^ Participants completed the VAS assessment of homeostatic drive to eat (hunger: “How hungry do you feel?”; fullness: “How full do you feel?”) at the following time points during the visit: (1) fasting, at the beginning of the visit; (2) fasting, before the meal; (3) immediately after the meal; (4) before the snack; and (5) after the snack, before the CTT (see SM 2 for a visual representation). Post-snack and before the CTT (SM 2), participants completed the VAS assessment of hedonic drive to eat (Figure 1), during which they answered the following questions by making a mark on a 100 mm line with extremes on either end: “How strong is your desire to eat your favourite food?” and “How satisfied are you?” The distance in mm from the left end of the line (0 mm = no desire at all/not at all satisfied) to the participant’s mark was then measured, yielding a score from 0 to 100.

Following the meal and snack, and after completion of the VAS assessment of hedonic drive to eat, the CTT was administered as a standardised test to assess hedonic eating behaviour.^32^ Participants were asked to taste an assortment of cookies, and rate them on various dimensions (e.g., sweetness, texture, and likability). They were instructed to sample as many cookies as they desired and needed to give their ratings. The quantity of cookies that each participant consumed and the corresponding caloric content served asa behavioural indicator of reward-related food intake.^34–36^

### 2.5 | Statistical analyses

Data were analysed using R software.^37^ Data distribution was assessed via the Shapiro–Wilk test. Non-normally distributed data were loge-transformed (OT levels at all measurement time points) or square root-transformed (VAS ratings) to achieve approximate normality. Descriptive statistics of the study sample are reported in Table 1 overall and per biological sex.

**FIGURE 2:**
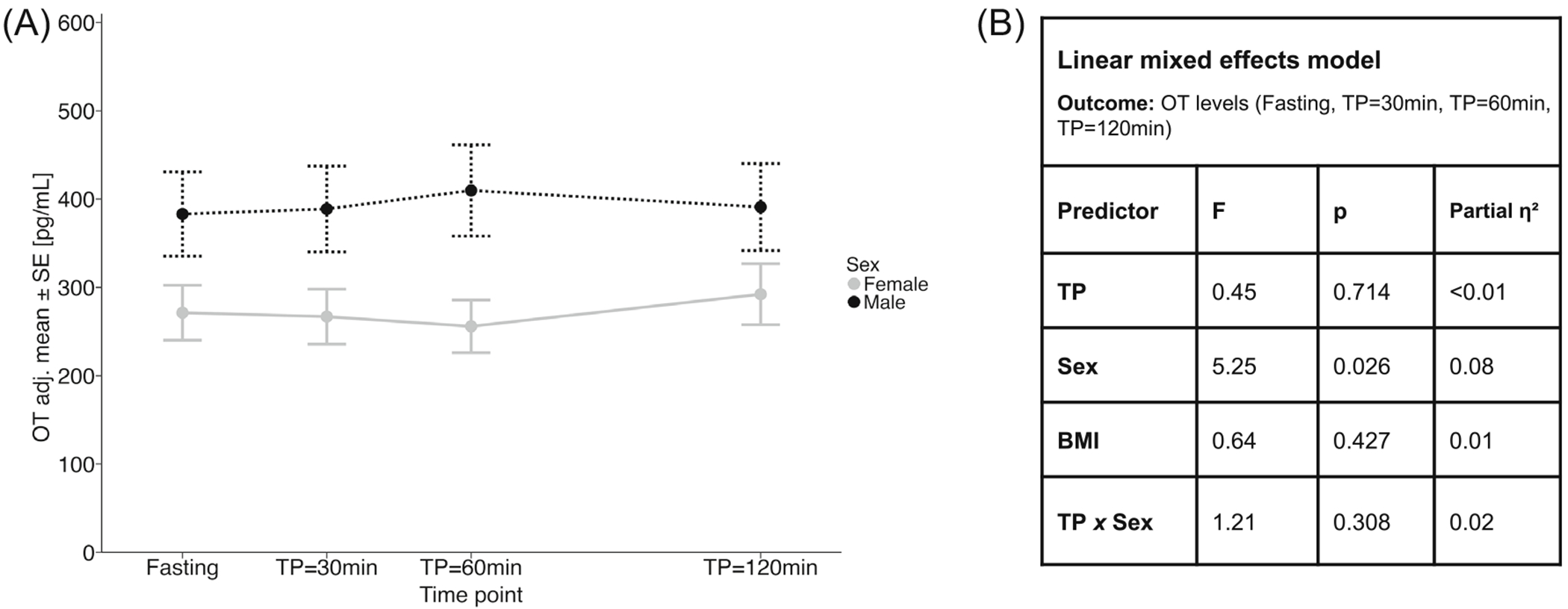
Meal-related OT dynamics. BMI, body mass index; OT, oxytocin; TP, time point. (A) Line plot of OT model estimates (back-transformed to original scale) around the standardised meal in female and male adults with obesity. Adjusted means and standard err ors (SE) are plotted for OT levels fasting and 30, 60, and 120 min after the standardised meal. (B) Estimates were obtained from a linear mixed effects model. OT levels were log_e_-transformed prior to analysis. Effects of TP, sex, and the TP-*x*-sex interaction were of interest, covarying for BMI. Model statistics are presented in ANOVA format.

We examined meal-related OT dynamics with a random-intercept linear mixed effects model with predictors TP, sex, and the TP-*x*-sex interaction, covarying for BMI. Model statistics are presented in ANOVA format (Figure 2). Given OT levels did not differ pre- versus postprandially (Figure 2), we computed the area under the curve with respect to ground (AUC) as a composite measure of OT exposure for subsequent analyses, integrating pre- and postprandial OT levels: OT AUC = ½*(OT_TP=fasting_ + OT_TP=30min_)*30 min + ½*(OT_TP=30min_ + OT_TP=60min_)*(60 min – 30 min) + ½*(OT_TP=60min_ + OT_TP=120min_)*(120 min – 60 min). OT AUC was computed in participants with available OT values at all pre- and postprandial time points (*n* = 51).

We then analysed associations of log_e_-transformed OT AUC with VAS ratings for hedonic drive to eat and caloric intake during the CTT using linear regression. Regression analyses were conducted in subsamples with complete data, that is, available VAS ratings (*n* = 41) and CTT caloric intake measures (*n* = 59; of these, *n* = 51 also had OT AUC data). Multiple testing adjustment was applied across both VAS hedonic appetite measures using the false discovery rate (FDR, Benjamini–Hochberg). Significant associations were followed up with a regression-based mediation model to investigate whether the relationship between OT AUC (independent variable, assessed first during visit) and hedonic eating behaviour measured by the CTT (dependent variable, assessed last) was mediated by subjective hedonic appetite measured via VAS (mediator, assessed between OT AUC and CTT). Finally, we explored potential effects of biological sex on the associations between OT AUC and measures of hedonic drive to eat.

Supplementary sensitivity analyses were conducted to assess the robustness of the associations between OT AUC and hedonic eating measures (VAS satisfaction and desire to eat favourite food ratings, CTT caloric intake). Specifically, linear regression models were additionally adjusted for (i) calories consumed during the standardised meal, (ii) calories consumed during the snack, and (iii) VAS fullness ratings following the meal and snack, thereby accounting for inter-individual variations in caloric intake and homeostatic fullness preceding the hedonic eating assessments (SM 4, models 1–9). Furthermore, we conducted an additional sensitivity analysis examining associations between fasting OT levels (instead of OT AUC) and both VAS ratings and CTT energy intake to explore more general links between OT and hedonic drive to eat that are independent of food intake-related mechanisms (SM 4, models 10–12).

## 3 | RESULTS

### 3.1 | Sample characteristics

Of the 61 study participants, 33 were female and 28 were male; the overall mean ± SE age was 33.6 ± 0.8 years (Table 1). The study sample was diverse regarding race and ethnicity. BMI was 36.8 ± 0.6 kg/m^2^ overall and did not differ between female and male participants. OT AUC was higher in male than female participants (*p* = 0.017, *d* = 0.70; Table 1). Overall caloric intake at the standardised mixed meal and snack was 466 ± 9 kcal and 189 ± 5 kcal, respectively, and similar across female and male participants, indicating that most of the provided food was consumed (Table 1).

Homeostatic hunger decreased while fullness increased following the standardised meal and snack (SM 2), as expected. Post-snack hunger was low (VAS 25.1 ± 3.7 out of 100 on average; Table 1, SM 2), allowing for assessment of hedonic drive to eat. Post-snack VAS ratings for desire to eat favourite food and satisfaction were 29.3 ± 4.0 and 61.5 ± 3.9 out of 100, respectively, in the overall sample and did not differ between female and male participants (Table 1). In contrast, caloric intake during the CTT was higher in male (293 ± 41 kcal) than in female (159 ± 19 kcal) participants (*p* = 0.005, *d* = 0.81; Table 1).

### 3.2 | Secretory OT dynamics surrounding a meal

We detected no differences in OT levels across meal-related time points (*p* = 0.714, partial η^2^ < 0.01; Figure 2). BMI (*p* = 0.427) did not significantly predict OT levels (Figure 2B). However, we identified a significant main effect of sex on OT levels around the meal (*p* = 0.026, partial η^2^ = 0.08): male participants had higher OT levels than female participants across all measurement time points (Figure 2A). However, the TP-*x*-sex interaction effect was not significant (*p* = 0.308, partial η^2^ = 0.02; Figure 2B). Given nonsignificant meal-related OT dynamics, the composite measure OT AUC was used for all subsequent analyses. Furthermore, BMI was omitted as a covariate in subsequent analyses, as it had no significant effect on OT levels.

### 3.3 | Relationships between OT exposure and hedonic drive to eat

**FIGURE 3:**
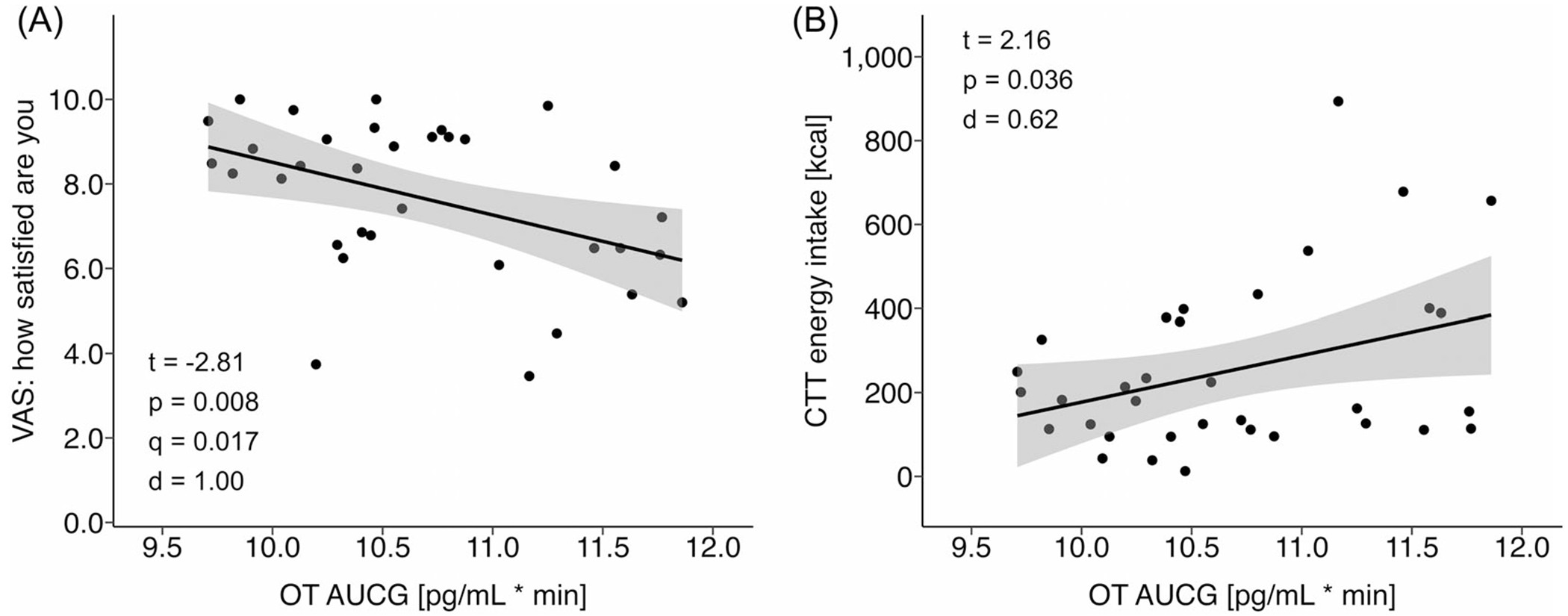
Associations between OT exposure around a meal and hedonic eating measures. AUCG, area under the curve with respect to ground; CTT, Cookie Taste Test; OT, oxytocin; VAS, visual analog scale. (A) Scatterplot with linear regression line illustrating the relationship between OT AUC and postprandial VAS satisfaction rating. Statistics are presented in the plot (*q* = FDR multiple testing adjustment of *p*-values was applied across regression models for VAS satisfaction and VAS desire to eat favourite food [not shown here/not significant]). Exploratively, a main effect of sex and an OT AUC-*x*-sex interaction effect were added to the regression model. Sex effects on VAS satisfaction ratings were not significant (sex: *T* = 1.17, *p* = 0.250, *d* = 0.43; OT AUC-*x*-sex: *T* = −1.24, *p* = 0.223, *d* = 0.45). (B) Scatterplot with linear regression line illustrating the relationship between OT AUC and CTT caloric intake. Statistics are presented in the plot. Exploratively, a main effect of sex and an OT AUC-*x*-sex interaction effect were added to the regression model. Sex effects on CTT caloric intake were not significant (sex: *T* = −1.29, *p* = 0.205, *d* = 0.38; OT AUC-*x*-sex: *T* = 1.42, *p* = 0.163, *d* = 0.41).

**FIGURE 4:**
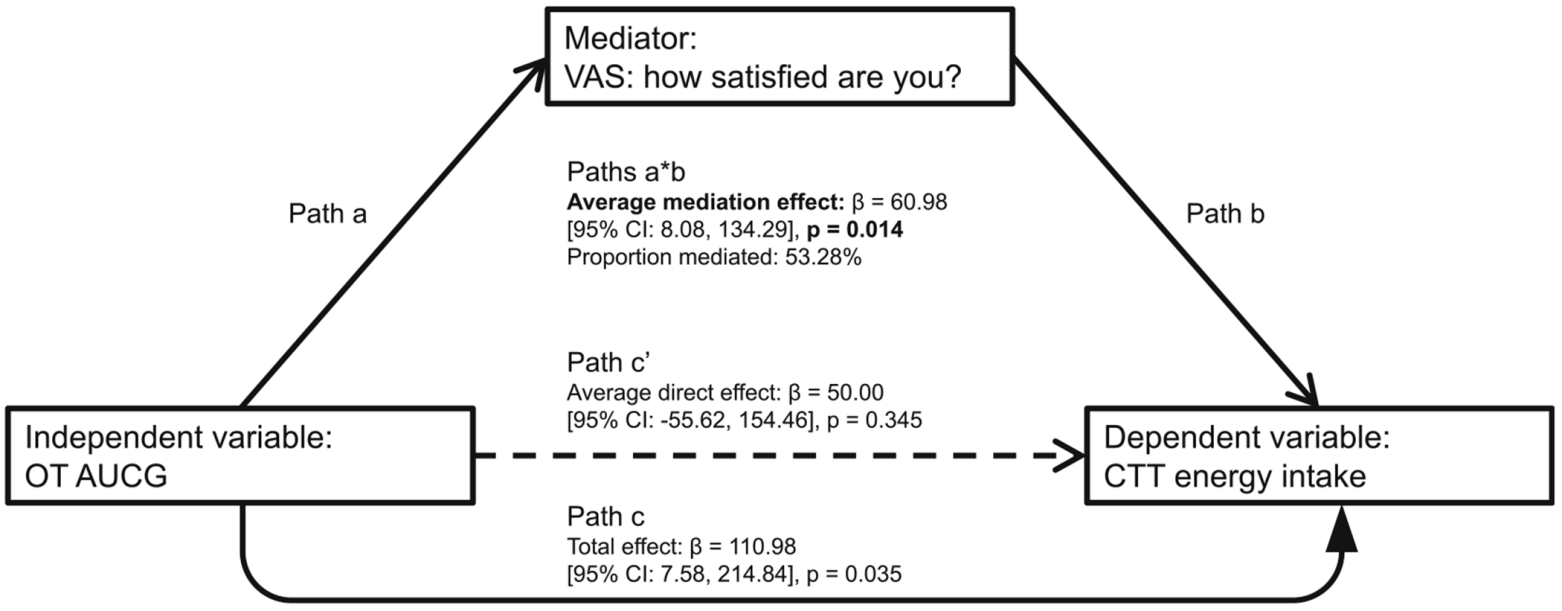
Mediation model results. AUCG, area under the curve with respect to ground; CTT, Cookie Taste Test; OT, oxytocin; VAS, visual analog scale. Regression-based mediation analysis was performed with 10 000 simulations and quasi-Bayesian confidence intervals. Proportion mediated (mediation effect divided by total effect) was computed as an effect size measure. During the study visits, the components of the mediation model were assessed in a temporally appropriate sequence: OT levels were measured, and OT AUC was calculated first (independent variable/predictor), followed by post-meal and post-snack VAS ratings (mediator), and finally the CTT (dependent variable/outcome).

OT AUC was inversely associated with post-snack hedonic satisfaction (*p* = 0.008, FDR-*q* = 0.017, *d* = 1.00), that is, higher OT exposure around a meal correlated with lower postprandial VAS satisfaction ratings (Figure 3A). In contrast, the association between OT AUC and VAS desire to eat favourite food was positive but only trend-level (*p* = 0.069, FDR-*q* = 0.069, *d*= 0.66). OT AUC significantly and positively predicted CTT caloric intake (*p* = 0.036, *d* = 0.62), that is, the higher the OT exposure surrounding a meal, the more calories were consumed during the CTT (Figure 3B). We did not detect effects of biological sex on the assessed associations between OT exposure around a meal and measures of hedonic eating drive (see Figure 3 legend). Notably, the relationship between OT AUC and CTT caloric intake was mediated by OT AUC’s effect on postprandial satisfaction (mediation effect: *p* = 0.014, proportion mediated = 53.3%; Figure 4).

Supplementary sensitivity analyses confirmed that the associations between OT AUC and VAS satisfaction ratings as well as CTT caloric intake remained significant after adjusting the regression models for calories consumed during the standardised meal and snack and for postprandial fullness following both (SM 4, models 1–9). An additional sensitivity analysis examining fasting OT concentrations instead of OT AUC revealed a significant positive association with CTT energy intake (*p* = 0.026, *d* = 0.60), comparable in strength to that observed for OT AUC (SM 4, model 12). In contrast, the association between fasting OT and VAS satisfaction reached only trend-level significance (*p* = 0.067, *d* = 0.61) but exhibited the same directionality as the relationship with OT AUC (SM 4, model 10).

## 4 | DISCUSSION

Contrary to our hypothesis, we observed no change in circulating OT levels in response to a standardised mixed meal in adults with obesity, suggesting a potential disruption in appetitive signalling. Higher OT levels measured around the meal predicted greater hedonic eating behaviour, consistent with our hypothesis, with this relationship mediated by reduced postprandial satisfaction, which is indicative of heightened subjective appetite. These findings enhance our understanding of endogenous OT physiology in human obesity and support a role for OT in modulating reward-driven eating beyond homeostatic needs—a factor that might contribute to the development of obesity and impede adherence to dietary interventions.

The absence of a postprandial OT change in the setting of human obesity contrasts with previous studies in normal-weight rodents, where plasma OT levels increased following food ingestion,^24^ and with human studies in healthy girls and women^26,27^ as well as in atypical anorexia nervosa,^28^ in which OT levels decreased postprandially. Similar to our findings in the setting of human obesity, circulating OT levels did not change in response to a standard meal in women with polycystic ovary syndrome (PCOS)^27^ and in two groups of individuals with restrictive eating disorders: youth with avoidant/restrictive food intake disorder^38^ and severely low-weight females with full-syndrome anorexia nervosa.^28^ Taken together, these studies, along with our current findings, suggest that meal-related OT dynamics may be disrupted in certain eating disorders, in endocrine disturbances such as hyperandrogenism and insulin resistance in PCOS, and in states of extreme deviations in energy balance such as obesity—pointing to a broader pattern of altered OT signalling across metabolic conditions. This interpretation is further supported by findings from diet-induced obese mice, which suggest a dysregulation in OT secretion patterns compared with chow-fed control mice.^39^ Consequently, food intake-related OT dynamics in human obesity—characterised by multiple metabolic and endocrine alterations, including dysregulation of appetite-regulating hormones^40^—may differ from those observed in healthy individuals and rodent models, potentially reflecting features specific to obesity pathophysiology.

The relationship between peripheral and central OT levels in the context of appetite regulation remains unclear. In adult rats, centrally administered alpha-melanocyte-stimulating hormone induced dendritic OT release from hypothalamic neurons in the supraoptic nucleus, while OT secretion into the bloodstream was inhibited by this intervention, resulting in decreased peripheral OT levels.^23^ Based on this reciprocal relationship observed in rodents, higher peripheral OT exposure might conversely indicate suppressed central availability and anorexigenic brain signalling of OT in humans, as well, leading to lower postprandial satisfaction and enhanced reward-driven eating behaviour. This interpretation would be in line with the directionality of our findings in adults with obesity. On average, they showed higher caloric intake during the CTT compared with normal-weight individuals reported in previous studies (223 kcal vs. 136–180 kcal).^31,36^ It is plausible that in obesity, peripheral OT levels inversely mirror central OT activity even in the absence of feeding, potentially leading to chronic alterations in the appetite-regulating neurocircuitry and contributing to the pathophysiology of obesity. Supplementary analyses using fasting peripheral OT concentrations instead of meal-related OT exposure yielded a similar positive association with CTT energy intake, suggesting that OT may broadly reflect hedonic eating tendencies in adults with obesity, independent of recent food intake. Although the relationship between fasting OT levels and postprandial satisfaction ratings only approached significance, its directionality mirrored that of the meal-related OT exposure association, highlighting the stability of our findings.

An alternative explanation for our findings is potential OT resistance in obesity, which could account for the observed correlation between higher OT exposure and greater hedonic eating—the opposite pattern to the relationship between OT levels around a meal and homeostatic appetite reported in healthy females^26,27^ and in females with atypical anorexia nervosa.^28^ Recent gain- and loss-of-function studies indicated distinct functioning of hypothalamic OT neuronal populations, with paraventricular nucleus OT neurons signalling meal termination and supraoptic nucleus OT neurons increasing meal size.^41^ As supraoptic neurons are thought to primarily reflect peripheral neuroendocrine signalling, the authors proposed that supraoptic magnocellular neurons may increase caloric intake via peripheral OT release. It is possible that greater tonic supraoptic OT neuronal activation in individuals with obesity leads to increased peripheral OT levels and orexigenic signalling.

Further animal and human research on obesity-specific fasting and postprandial OT alterations in relation to hedonic drive to eat will be essential to determine the complex interplay of specific central and peripheral OT circuits in appetitive behaviours and delineate core mechanisms that may underlie the development and persistence of human obesity. Importantly, our mediation findings suggest a potential mechanism by which endogenous OT influences hedonic eating behaviour—namely, through its association with reduced postprandial subjective satisfaction, which may indirectly reflect increased hedonic appetite.

We also explored sex differences in endogenous OT dynamics and their associations with hedonic appetite and eating behaviour, given that OT is a sexually dimorphic hormone.^42^ While studies evaluating OT levels between the two sexes generated conflicting results,^29,43,44^ we found that women with obesity had consistently lower OT levels than men while fasting and in response to a standardised meal (Figure 2). Oestrogen stimulates OT production,^45^ while testosterone has been shown to suppress central OT gene expression.^42^ Normal-weight women were previously found to have significantly higher peripheral OT levels than men.^43^ Conversely, the higher OT levels that we observed in men compared to women with obesity may be linked to testosterone aromatization to oestrogen in the adipose tissue.^46^ In addition, increased androgen production in women with obesity^47,48^ might also contribute to this observation.

A strength of our study is the robustness of the observed associations between OT exposure and measures of hedonic drive to eat. Sensitivity analyses demonstrated that the relationships between OT AUC and both VAS satisfaction ratings and CTT caloric intake remained consistent even after controlling for caloric intake during the standardised meal and snack, as well as postprandial fullness. This indicates that these associations are unlikely to be driven by individual differences in prior energy consumption or homeostatic satiety.

Study limitations include the relatively small sample size. For example, while our analyses did not reveal significant sex differences in the association between OT exposure and hedonic drive to eat, such effects may become evident in a larger-scale study with greater statistical power. Furthermore, power was reduced due to missing data, particularly for postprandial OT measurements and VAS ratings. Missingness arose from unsuccessful or compromised blood draws, samples not meeting OT assay standards, early termination of study visits, and, for postprandial VAS ratings, occasional de-prioritisation of assessments due to time constraints. Additionally, OT levels were not measured earlier than 30 min post-meal. We cannot exclude the possibility that a transient OT spike occurred within the first few minutes after food intake and had already declined by the time of the 30-min assessment. Furthermore, OT assays and detection techniques still vary, particularly in their ability to measure protein-bound OT and OT fragments.^49^ Thus, OT concentrations obtained using different methods are not directly comparable.^50^ Nevertheless, existing assays allow for comparisons of relative differences in peripheral OT levels between groups or across time points. In our prior work, we demonstrated consistent findings across clinical populations using different assay methods and both extracted and unextracted samples, including the use of ELISA in unextracted plasma, as applied in the present study.^50^ Finally, as our data were derived from a randomised clinical trial investigating OT as a weight-loss therapeutic in adults with obesity,^16,30^ no control group of normal-weight individuals was available. Examining OT dynamics and their associations with hedonic eating in individuals with versus without obesity could be a promising avenue for future research.

In conclusion, this study demonstrates that circulating OT levels in adults with obesity do not change in response to a standardised meal. However, higher integrated OT concentrations around the meal—likely reflecting basal OT tone—predict increased hedonic eating, an effect mediated by increased subjective appetite. These findings underscore the importance of further investigations of endogenous OT function and its appetite-regulating mechanisms across weight statuses and eating disorders to inform OT-targeted therapeutic strategies.

## Supplementary Material

Supplements

Additional supporting information can be found online in the Supporting Information section at the end of this article.

## Figures and Tables

**TABLE 1:** Descriptive statistics.

Characteristic	Overall sample (*n* = 61)		Female participants (*n* = 33, 54.10%)	Male participants (*n* = 28, 45.90%)	Test statistics		
	Mean ± SE/count (%)	Range (min. - max.)	Mean ± SE /count (%)	Mean ± SE /count (%)	*t*	*p*	*d*
Age (years)	33.55 ± 0.81	18.60–45.71	33.16 ± 1.16	34.00 ± 1.13	−0.52	0.607	0.13

Race							
Asian	6 (9.84%)	n/a	3 (9.09%)	3 (10.71%)	n/a	n/a	n/a
Black/African American	14 (22.95%)	n/a	9 (27.27%)	5 (17.86%)			
White	38 (62.30%)	n/a	20 (60.61%)	18 (64.29%)			
More than one race	2 (3.28%)	n/a		2 (7.14%)			
None of the above	1 (1.64%)	n/a	1 (3.03%)				

Ethnicity							
Hispanic/Latino ethnic group	13 (21.31%)	n/a	8 (24.24%)	5 (17.86%)	n/a	n/a	n/a

Days since last menstrual period	n/a	n/a	13.94 ± 1.63	n/a	n/a	n/a	n/a

Anthropometrics							
Body weight (kg)	109.02 ± 2.48	76.50–165.00	102.95 ± 3.01	116.17 ± 3.68	−2.78	0.007	0.72
BMI (kg/m^2^)	36.77 ± 0.62	30.20–50.60	37.42 ± 0.86	35.99 ± 0.88	1.16	0.251	0.30

OT AUC_G_ (pg/mL*min)	49077.91 ± 4720.01	16450.35 – 141 570	38799.06 ± 4797.14	59767.91 ± 7772.62	−2.48	0.017	0.70

Calories consumed at the standardised meal (kcal)	465.69 ± 9.29	167.63–554.05	461.75 ± 9.35	470.06 ± 16.78	−0.43	0.667	0.12

Calories consumed at the standardised snack (kcal)	189.28 ± 5.09	71.92–214.40	184.38 ± 7.34	194.92 ± 6.95	−1.04	0.302	0.27

Post-snack VAS (0–100) ratings							
Hunger	25.05 ± 3.66	0–76	17.14 ± 4.30	33.35 ± 5.50	−2.14	0.038	0.67
Fullness	61.68 ± 4.09	5–100	69.76 ± 5.26	53.20 ± 5.84	1.88	0.067	0.59
Desire to eat favourite food	29.29 ± 4.03	0–85	24.57 ± 5.18	34.25 ± 6.16	−1.10	0.276	0.35
How satisfied are you	61.49 ± 3.86	12–100	67.00 ± 5.18	55.70 ± 5.59	1.41	0.168	0.44

CTT energy intake (kcal)	222.56 ± 23.24	12.69–893.65	158.76 ± 18.75	293.19 ± 40.76	−3.00	0.005	0.81

*Note*: Sex differences were tested with two-sample *t*-tests. As test statistics, mean ± standard error of the mean (SE), range (minimum–maximum, in the overall sample), *t*-value, *p*-value, and effect size estimate Cohen’s *d* are stated. OT AUC and VAS ratings are presented in their original scale but were transformed (log_e_ for OT AUC, sqrt for VAS ratings) prior to analysis.

Abbreviations: AUC_G_, area under the curve with respect to ground; BMI, body mass index; CTT, Cookie Taste Test; OT, oxytocin; VAS, visual analog scale.

## Data Availability

The data that support the findings of this study are not publicly available due to patient privacy and confidentiality requirements. De-identified data may be made available from the corresponding author upon reasonable request and with appropriate institutional and ethical approvals.
