# Supplementary material for: Oxytocin levels do not change around a meal and correlate with reward-driven caloric consumption in adults with obesity
Source: Diabetes Obes Metab. Author manuscript; Available in PMC 2026 May 18. (PMC13182910; doi:10.1111/dom.70340)
Supplement: Supplements [file NIHMS2166236-supplement-Supplements.docx]

**Supplementary Materials to Wronski & Boutin et al.**

**SM 1: Exclusion criteria for the parent study (ClinicalTrials.gov NCT03043053)**

A. Substance use disorder active within the last 6 months, or clinical suspicion of ongoing substance use disorder at the discretion of the study clinician at the time of screening based on history and/or lab results. B. Medication changes within 4 weeks of enrollment. C. Use of prescription or over the counter drugs or dietary/herbal supplements. D. History of any of the following medical conditions: cardiovascular disease, including prolonged QT; clinically significant gastrointestinal disorders; bariatric surgery (except for those participants with a history of laparoscopic adjustable gastric band surgery); bleeding disorder; epilepsy; untreated thyroid disease. E. History of an eating disorder or psychosis; current psychiatric disorder unless on stable dose of anti-depressant or anxiolytic treatment for 6 months or more, no findings for active symptoms that would qualify for an active psychiatric diagnosis as assessed by the Mini International Neuropsychiatric Interview (M.I.N.I.), and a normal QT on EKG at the screening visit. F. Hematocrit >2% below normal. G. Hemoglobin a1c ≥6.5%. H. ALT or AST >2.5 times upper limit of normal. I. Cr >1.5 mg/dL. J. Hyponatremia. K. Pregnancy or breastfeeding. L. Unwilling to use a medically acceptable form of contraception throughout the study period (females of child-bearing potential only). M. Weight change >5 kg within 3 months prior to randomization. N. Follows a nonstandard diet (Paleo, Atkins, raw diet, macrobiotic diet). O. Significant change in physical activity or diet within 1 month of randomization. P. Malignancy within 5 years of screening visit (except basal cell carcinoma or squamous cell carcinoma). Q. Participation in any clinical study involving an investigational drug, device, or biologic within 1 month of randomization. R. Current smoking or tobacco use. S. Contraindications to MRI and/or DXA (e.g., MRI: weight >440 lbs and maximum body circumference >170 cm; DXA: weight >350 lbs). T. Allergy to lidocaine.

**SM 2: Visual analog scale hunger and satiety ratings during the study visit**

*Abbreviations: TP, time point; VAS, visual analog scale.*

Line plots illustrate how homeostatic hunger and fullness developed in relation to food intake during the study visit. Participants performed assessments of hedonic drive to eat (VAS desire to eat favorite food, VAS satisfaction, Cookie Taste Test) after the standardized meal and snack, when homeostatic hunger was low and satiety high. Thus, hedonic drive to eat could be evaluated.

**SM 3: Cookie Taste Test energy intake data visualization**

*Abbreviations: CTT, Cookie Taste Test.*

Box plots illustrate the distribution of CTT energy intake in female and male participants (box: 50% of data from Q1 to Q3; whiskers: Q1 - 1.5*IQR, Q3 + 1.5*IQR; cross: mean). There were three outlier occurrences (> Q3 + 1.5*IQR; n=2 females, n=1 male).

**SM 4: Sensitivity analyses for associations between OT and hedonic eating measures**

|  | **Regression model** | **Predictor/covariate** | **t** | **p** | **q** | **d** | **Main model results confirmed?** |
| --- | --- | --- | --- | --- | --- | --- | --- |
| 1 | VAS satisfaction ~ OT AUC + standardized meal intake | **OT AUC** | -2.92 | 0.007 | 0.013 | 1.07 | yes |
|  |  | Standardized meal intake | 0.72 | 0.479 | 0.958 | 0.26 |  |
| 2 | VAS desire to eat favorite food ~ OT AUC + standardized meal intake | **OT AUC** | 2.04 | 0.050 | 0.050 | 0.74 | yes |
|  |  | Standardized meal intake | 0.00 | 0.999 | 0.999 | 0.00 |  |
| 3 | CTT energy intake ~ OT AUC + standardized meal intake | **OT AUC** | 2.11 | 0.040 | n/a | 0.61 | yes |
|  |  | Standardized meal intake | 0.05 | 0.962 | n/a | 0.01 |  |
| 4 | VAS satisfaction ~ OT AUC + snack intake | **OT AUC** | -2.81 | 0.009 | 0.017 | 1.03 | yes |
|  |  | Snack intake | 0.46 | 0.652 | 0.981 | 0.17 |  |
| 5 | VAS desire to eat favorite food ~ OT AUC + snack intake | **OT AUC** | 2.03 | 0.051 | 0.051 | 0.74 | yes |
|  |  | Snack intake | -0.02 | 0.981 | 0.981 | 0.01 |  |
| 6 | CTT energy intake ~ OT AUC + snack intake | **OT AUC** | 2.40 | 0.020 | n/a | 0.70 | yes |
|  |  | Snack intake | 1.60 | 0.117 | n/a | 0.47 |  |
| 7 | VAS satisfaction ~ OT AUC + VAS fullness (fed state) | **OT AUC** | -2.39 | 0.023 | 0.047 | 0.87 | yes |
|  |  | VAS fullness | 3.36 | 0.002 | 0.004 | 1.23 |  |
| 8 | VAS desire to eat favorite food ~ OT AUC + VAS fullness (fed state) | **OT AUC** | 1.56 | 0.130 | 0.130 | 0.57 | yes |
|  |  | VAS fullness | -2.36 | 0.025 | 0.025 | 0.86 |  |
| 9 | CTT energy intake ~ OT AUC + VAS fullness (fed state) | **OT AUC** | 2.24 | 0.033 | n/a | 0.82 | yes |
|  |  | VAS fullness | 0.77 | 0.445 | n/a | 0.28 |  |
| 10 | VAS satisfaction ~ fasting OT (T0) | **OT T0** | -1.89 | 0.067 | 0.133 | 0.61 | no (trend-level) |
| 11 | VAS desire to eat favorite food ~ fasting OT (T0) | **OT T0** | 0.55 | 0.586 | 0.586 | 0.18 | yes |
| 12 | CTT energy intake ~ fasting OT (T0) | **OT T0** | 2.28 | 0.026 | n/a | 0.60 | yes |

*Abbreviations: AUC, area under the curve (with respect to ground); CTT, Cookie Taste Test; OT, oxytocin; VAS, visual analog scale.*

Models 1-9: supplementary linear regression models assessing the relationship between OT AUC and postprandial VAS satisfaction ratings, VAS hedonic drive to eat ratings, and CTT energy intake, additionally adjusting/covarying for intake at the standardized meal (models 1-3), snack intake (models 4-6), and VAS fullness ratings post-meal and post-snack (models 7-9). Models 10-12: supplementary linear regression models assessing the relationship between fasting OT levels and postprandial VAS satisfaction ratings, VAS hedonic drive to eat ratings, and CTT energy intake. The last column indicates whether main model results (see main article, no covariate adjustment) were confirmed in these sensitivity analyses.
